# International development of a patient-centered core outcome set for assessing health-related quality of life in metastatic breast cancer patients

**DOI:** 10.1007/s10549-022-06827-6

**Published:** 2023-01-20

**Authors:** K. M. de Ligt, B. H. de Rooij, E. Hedayati, M. M. Karsten, V. R. Smaardijk, M. Velting, C. Saunders, L. Travado, F. Cardoso, E. Lopez, N. Carney, Y. Wengström, A. Ives, G. Velikova, M. D. L. Sousa Fialho, Y. Seidler, T. A. Stamm, L. B. Koppert, L. V. van de Poll-Franse, the Innovative Medicines Initiative - Health Outcomes Observatory (H2O) consortium

**Affiliations:** 1 Division of Psychosocial Research and Epidemiology, The Netherlands Cancer Institute, PO Box 90203, 1006 BE Amsterdam, The Netherlands; 2 Department of Research and Development, Netherlands Comprehensive Cancer Organisation, Utrecht, The Netherlands; 3 Department of Medical and Clinical Psychology, Center of Research on Psychological and Somatic Disorders (CoRPS), Tilburg University, Tilburg, The Netherlands; 4 Department of Oncology-Pathology, Bioclinicum, Karolinska Institutet, Solna, Stockholm, Sweden; 5 Breast Cancer Center, Cancer Theme, Karolinska University Hospital and Karolinska Comprehensive Cancer Centre, Stockholm, Sweden; 6 Department of Gynecology with Breast Center, Charité - Universitätsmedizin Berlin, Berlin, Germany; 7 Freie Universität Berlin and Humboldt-Universität zu Berlin, Berlin, Germany; 8 Dutch Breast Cancer Patient Association (Borstkankervereniging Nederland), Utrecht, The Netherlands; 9 Department of Surgery, Melbourne Medical School, Melbourne, Australia; 10 Breast Unit, Champalimaud Clinical Center/Champalimaud Foundation, Lisbon, Portugal; 11 Department of Radiation Oncology, Vithas Hospital La Milagrosa, GenesisCare, Madrid, Spain; 12 F.Hoffmann-La Roche Ltd, Basel, Switzerland; 13 Department of Oncology, Karolinska University Hospital, Stockholm, Sweden; 14 Cancer and Palliative Care Research and Evaluation Unit, University of Western Australia, Crawley, WA Australia; 15 Leeds Institute of Medical Research at St James’s, St James’s University Hospital, University of Leeds, Leeds, UK; 16 Leeds Cancer Centre, Leeds Teaching Hospitals NHS Trust, St James’s University Hospital, Leeds, UK; 17 International Consortium for Health Outcomes Measurement UK, London, UK; 18 Section for Outcomes Research, Center for Medical Statistics, Informatics and Intelligent Systems, Medical University of Vienna, Vienna, Austria; 19 Ludwig Boltzmann Institute for Arthritis and Rehabilitation, Vienna, Austria; 20 Department of Surgical Oncology, Erasmus MC Cancer Institute, Rotterdam, The Netherlands

**Keywords:** Metastatic breast cancer, Outcome measures, Health-related quality of life

## Abstract

**Purpose:**

For patients living with metastatic breast cancer (MBC), achieving best possible health-related quality of life, along with maximizing survival, is vital. Yet, we have no systemic way to determine if we achieve these goals. A Core Outcome Set (COS) that allows standardized measurement of outcomes important to patients, but also promotes discussing these outcomes during clinical encounters, is long overdue.

**Methods:**

An international expert group (EG) of patient advocates, researchers, medical specialists, nurse specialists, and pharmaceutical industry representatives (*n* = 17) reviewed a list of relevant outcomes retrieved from the literature. A broader group (*n* = 141: patients/patient advocates (*n* = 45), health care professionals/researchers (*n* = 64), pharmaceutical industry representatives (*n* = 28), and health authority representatives (*n* = 4)) participated in a modified Delphi procedure, scoring the relevance of outcomes in two survey rounds. The EG finalized the COS in a consensus meeting.

**Results:**

The final MBC COS includes 101 variables about: (1) health-related quality of life (HRQoL, *n* = 26) and adverse events (*n* = 24); (2) baseline patient characteristics (*n* = 9); and (3) clinical variables (*n* = 42). Many outcome that cover aspects of HRQoL relevant to MBC patients are included, e.g. daily functioning (including ability to work), psychosocial/emotional functioning, sexual functioning, and relationship with the medical team.

**Conclusion:**

The COS developed in this study contains important administrative data, clinical records, and clinician-reported measures that captures the impact of cancer. The COS is important for standardization of clinical research and implementation in daily practice and has received accreditation by the International Consortium for Health Outcomes Measurement (ICHOM).

**Supplementary Information:**

The online version contains supplementary material available at 10.1007/s10549-022-06827-6.

## Background

Breast cancer is the most commonly diagnosed cancer and leading cause of cancer death in women worldwide, with an estimated 2.2 million new cases and 685 thousand deaths in 2020 [1]. In high-income countries, 5–10% of patients present with metastatic breast cancer (MBC) at the time of initial diagnosis [2, 3]; 20–30% of primary breast cancer patients develop MBC over time [4, 5]. Five-year survival in early breast cancer is high (99% for localized, 83–86% for regional breast cancer) [3, 6], but remains poor in MBC patients (25–34%); median survival is estimated at 2–3 years [3, 5, 7]. As MBC remains incurable, treatment focusses on extending survival, controlling disease progression and associated symptoms, and improving or maintaining health-related quality of life (HRQoL) [8].

The MBC disease trajectory has been described as one of highs and lows [5, 9], where disease control and progression, fear and hope, and better and worse HRQoL constantly alternate. Adverse events (i.e. disease symptoms and treatment side effects) often reduce HRQoL. Therefore, disease status and treatment choice should be balanced with controlling adverse events, maximizing HRQoL, and respecting patients’ priorities and life plans [8]. Because MBC progresses, decisions are time-sensitive and patients face uncertain outcomes [5]. Adverse events management aims to avoid disrupting the patient’s activities of daily living, maintaining or restoring HRQoL, and continuing therapy for as long as needed. Adverse events should be followed systematically, and monitored regularly over time, to avoid serious, potentially fatal, adverse events that burden patients and could increase cost of care [8].

Patients struggle with the fact that they will not be cured [5, 10, 11]. The Advanced Breast Cancer International Consensus Guidelines (ABC-5) stress that patients should be offered psychosocial and supportive care and symptom-related interventions, from the time of MBC diagnosis. ABC-5 strongly recommends implementing patient-reported outcome measures (PROMs) in clinical care to record adverse events and allow personalized care [8].

For MBC patients, achieving the best possible HRQoL, along with maximizing survival, is essential [10]. Yet, we have no systematic way to determine if these goals are achieved. A Core Outcome Set (COS), allowing standardized measurement of outcomes most important to patients, is long overdue. For early breast cancer, the International Consortium for Health Outcomes Measurement (ICHOM) developed a COS recording survival and cancer control, adverse events, HRQoL, and case-mix factors through combined administrative, clinical, and PROMs data [12]. However, this COS is less relevant here, as the disease trajectory and experience of MBC patients is so distinct from primary breast cancer [5]. Therefore, we aimed to develop a COS applicable to, and capturing the perspective of, patients with MBC at first diagnosis or who developed it after early breast cancer treatment, designed to be incorporated into healthcare decision-making at individual and population level, in clinical practice and clinical research.

## Materials and methods

**Fig. 1:**
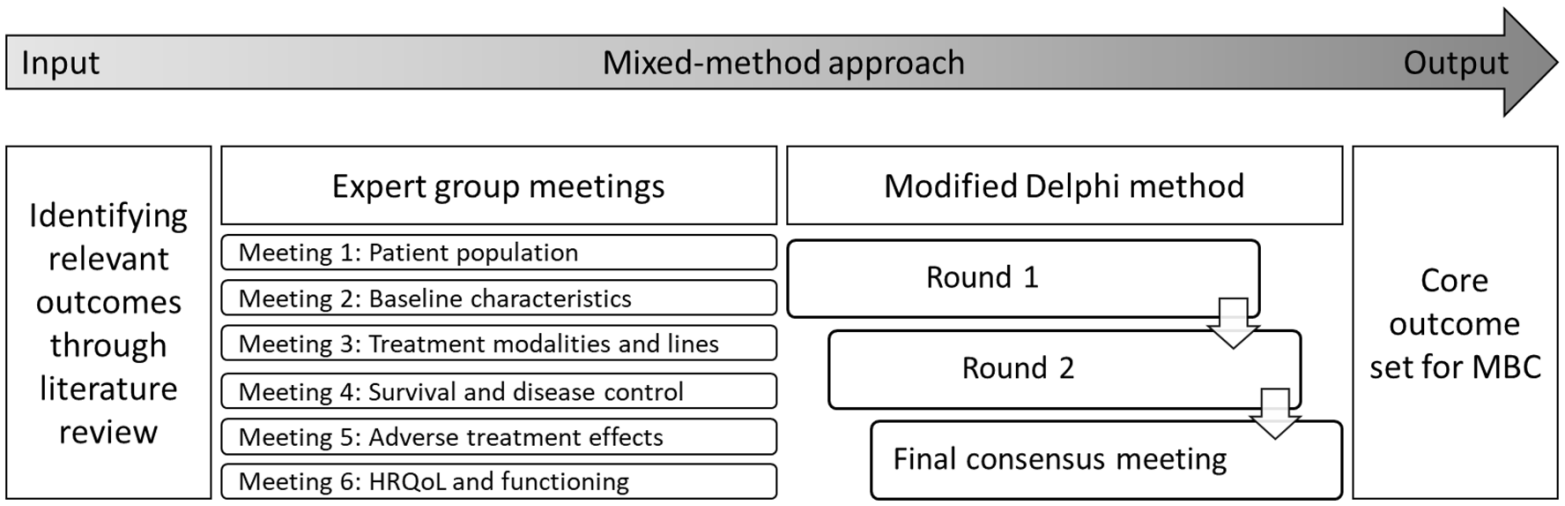
Mixed-method approach for developing core outcome set for patients with metastatic breast cancer. *HRQoL***:** Health-related quality of life, *MBC* Metastatic breast cancer

Our study used a mixed-method approach including literature review, expert group (EG) meetings, modified Delphi procedure, and final consensus meeting (Fig. 1), in line with The Core Outcome Measures in Effectiveness Trials (COMET) Handbook (version 1.0) for developing a COS [13]. The protocol was registered online [14].

### Identifying relevant outcomes

A potential outcomes list was generated from literature reviews conducted in two recent studies and searching the COMET database [15]. The semi-systematic review by Clarijs et al. [16] provided an overview of PROMs applied in MBC research. Outcomes were retrieved from the reported PROMs. The systematic review by Bedding et al. [17] was part of the development of a PROMs survey module for assessing HRQoL in MBC patients, in a project ran by the European Organisation for Research and Treatment of Cancer (EORTC) and the ABC Global Alliance. Adverse events and issues impacting HRQoL were noted.

### Expert group (EG) meetings

To review the identified outcomes, relevant experts were approached through ‘snowball sampling’ with a maximum variation strategy regarding age, expertise and geographical area. This included patient advocates, oncologists, surgeons, radiation oncologists, nurse specialists, and researchers. Between February and May 2021, six bi-weekly meetings discussed: (1) definition of patient population, (2) baseline characteristics, (3) treatment modalities/lines, (4) survival and disease control, (5) adverse events, (6) HRQoL and functioning. Before meetings, participants scored the relevance of outcomes on a 9-point Likert scale (1–3: ‘not that important’, 4–6: ‘important but not critical’, 7–9: ‘critically important’), based on the Grading of Recommendations, Assessment, Development and Evaluations (GRADE) method [18]. Following the COMET Handbook [13], the consensus threshold was set at > 50% of respondents scored the outcome 7–9 AND ≤ 15% in each group scored 1–3. Outcomes for which no consensus was reached or participants commented on, and newly suggested outcomes, were discussed and voted for (by counting all meeting participants in favor for including an outcome). A new list was constructed as input for the modified Delphi.

### Modified Delphi consensus procedure

A broader group of international patients/patient advocates, healthcare professionals (HCPs), academic researchers, pharmaceutical industry representatives, and health authority representatives were invited to participate in a modified Delphi. Invitations were sent out by the EG to the MBC working field through snowball sampling and to patients through patient associations or direct recruitment by HCPs in Austria, The Netherlands, Spain, Sweden and Portugal. In a modified Delphi, summarized feedback presented during the survey rounds replaces the meetings between survey rounds in a classical RAND Delphi [19–22]. We aimed to include 25–50 participants per stakeholder group [23]. The Delphi was available in English, Dutch, German, Spanish, Swedish, Portuguese, and managed through DelphiManager (COMET, 2016 [24]). Outcomes for each category were presented in non-random order, each outcome accompanied by a lay definition. Three patient advocates reviewed and pilot-tested the Delphi for comprehensiveness and completeness. The Netherlands Cancer Institute Institutional Review Board (IRB) declared that formal approval from an ethics committee was not required. Local execution was approved under registration number IRBd21-148. All participants gave electronic informed consent for participating in the Delphi.

In two survey rounds in September and October 2021, participants scored the relevance of outcomes from the preliminary list (GRADE 9-point Likert scale) [18] and could provide a reason for their scores or ‘unable to score’. Outcomes were included if ≥ 70% participants in each stakeholder group scored 7–9 (‘highly relevant’ AND ≤ 15% in each group scored 1–3 (‘less relevant’) [13]. Each round was open for two weeks. E-mail reminders were sent to non-responders after one week.

In the first round, participants’ background information was collected (age, sex, home country). Patient advocates could also add additional outcomes. These were reviewed by the EG and added to the second round if considered unique and additionally relevant.

In the second round, the summarized results from the first round were presented as histograms. Participants could change their scores (“reflect and re-rate”). The summarized responses served as input for the final consensus meeting.

### Final consensus meeting

The Delphi was concluded with three virtual EG sessions through Microsoft Teams in November and December 2021, with the following goals for each session: session (1) finalizing the COS; session (2) determining measurement frequencies; session (3) selecting outcome measures [13]. Outcomes were included if ≥ 70% of participants per stakeholder group scored 7–9 (‘highly relevant’) AND ≤ 15% in each group scored 1–3 (‘less relevant’). Since the COS aims to capture the patients’ perspective of cancer, outcomes rated important by patients were included immediately. While discussing potential outcomes, we considered the availability of data and challenges with measurement.

The defined COS was e-mailed to the group for final confirmation.

## Results

**Fig. 2:**
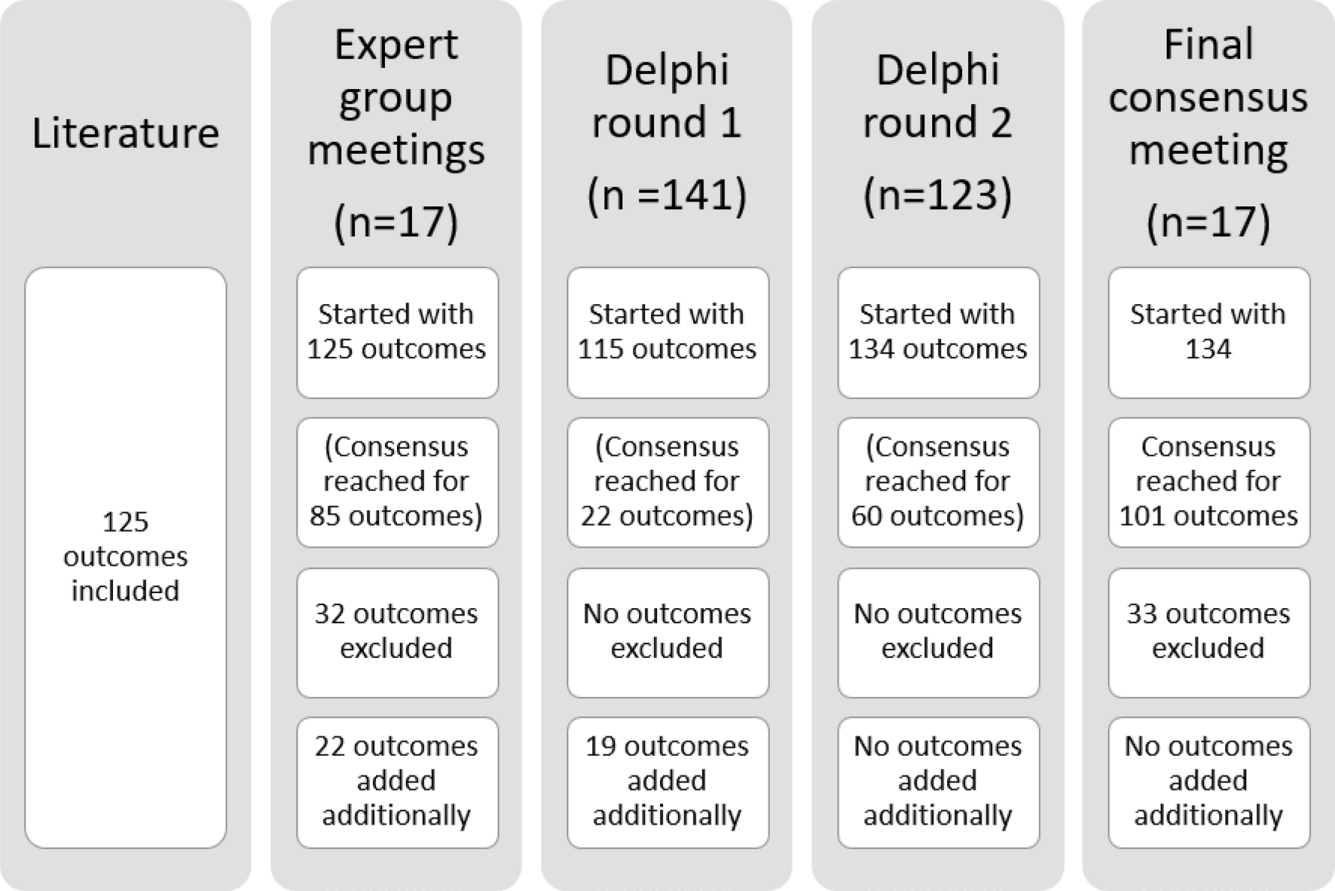
Participants and results per methodological round

Results from each methodological step are presented in Table 1 and Fig. 2.

**Table 1:**
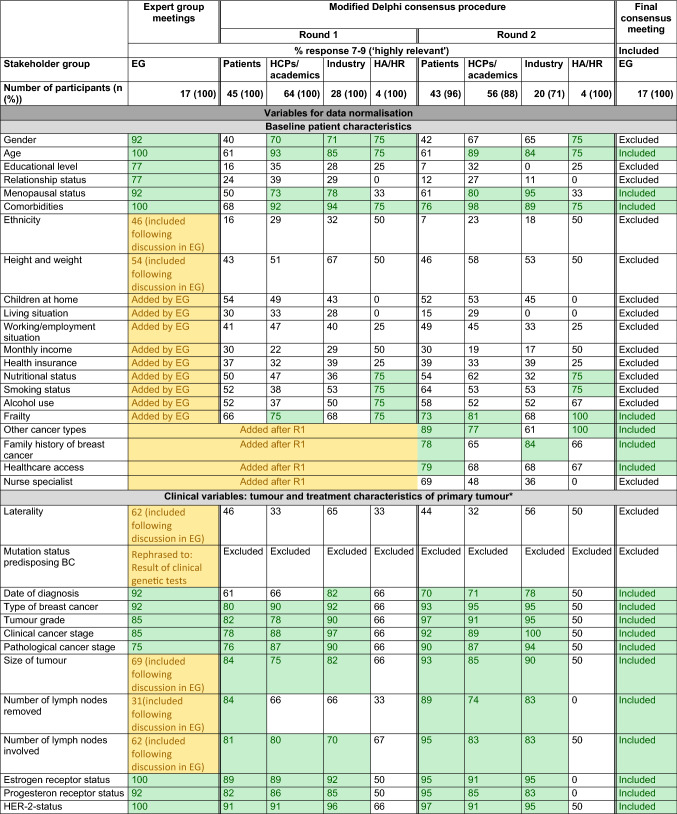

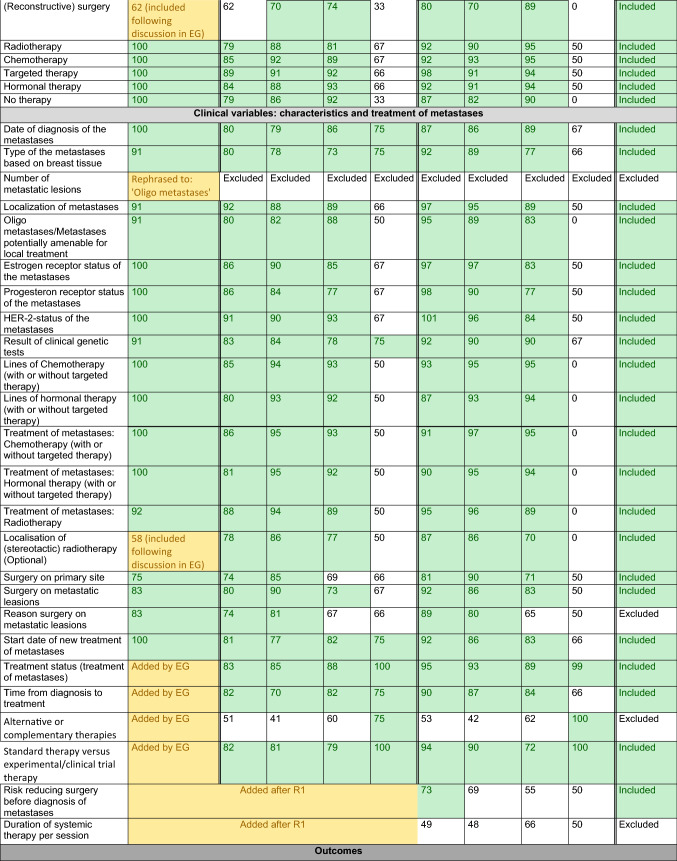

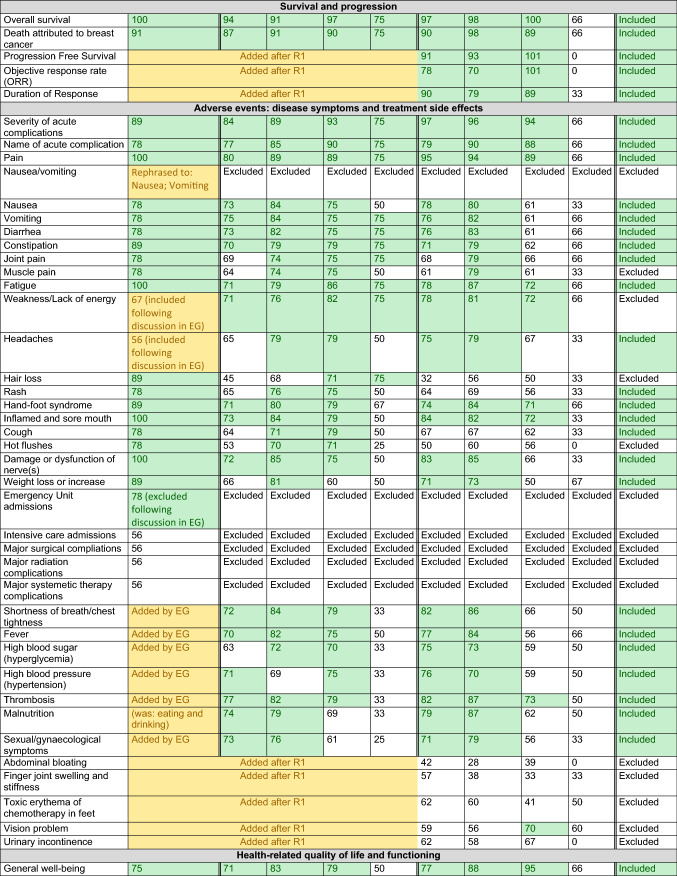

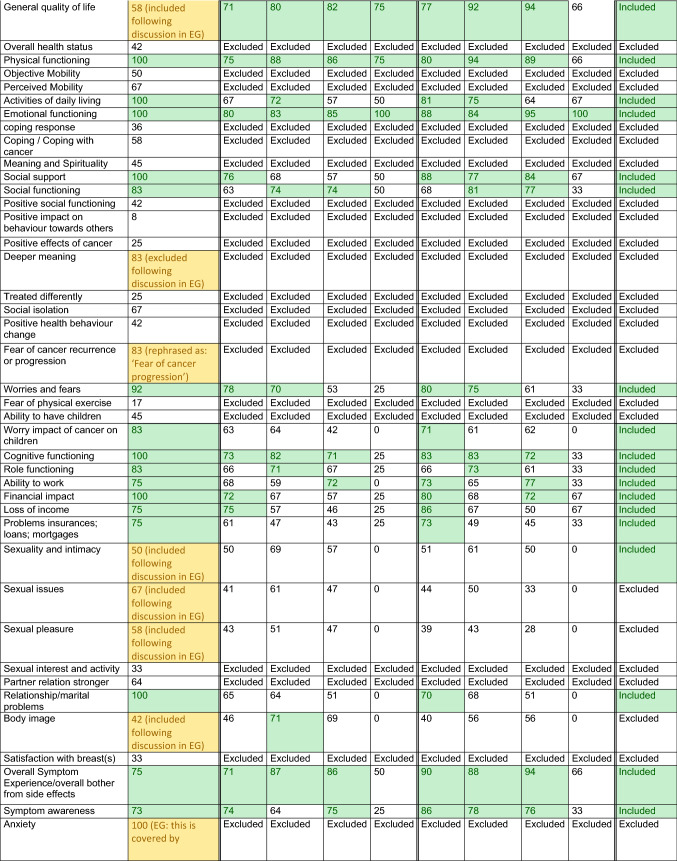

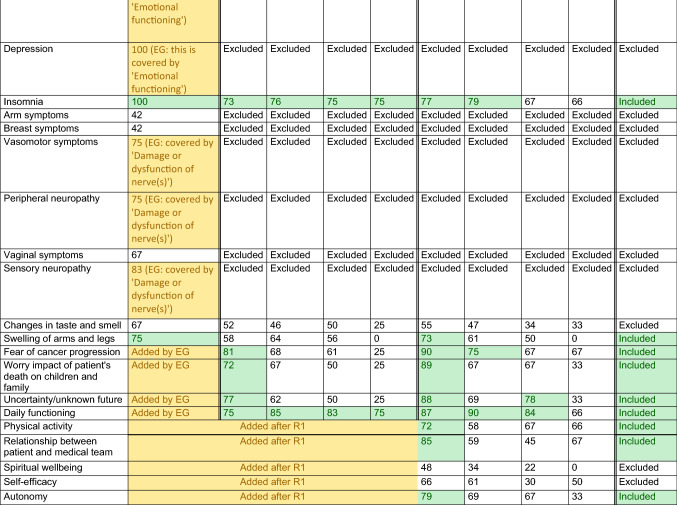
Relevance scores for each preliminary outcome per methodological round

*Description*: Each cell presents the percentage of participants who scored an item with relevance score 7–9 (‘highly relevant’); the final column concludes whether an item was eventually included in the COS

*Legend*: Green: consensus ≥ 70, outcome was transferred to next methodological round or included in final COS. Yellow: outcome was originally not included in the provisional outcomes list retrieved from the literature and therefore added during one of the methodological rounds.

^*^In case of metastases developed after initial treatment for early breast cancer (i.e. metachronous metastases), the diagnostic and treatment characteristics of the primary tumour are registered as well

*COS* core outcome set, *EG* expert group, *HA/HR* health authority/health regulator, *N/A* not applicable, outcome not included (in this methodological round or in the final COS), *R1* Delphi round 1

### Identifying relevant outcomes

We retrieved 125 outcomes, serving as input for the EG meetings (Table 1, second column).

Seventeen unique PROMs were identified, including the EORTC QLQ-C30 and QLQ-BR23, SF-36, FACT-B, and EQ-5D, illustrating the large heterogeneity of PROMs applied in clinical trials [16].

Commonly reported symptoms in included studies: gastrointestinal issues (diarrhoea, nausea, vomiting), muscular-skeletal issues (arthralgia, myalgia), skin issues (alopecia, rash, hand–foot syndrome), psychological issues (depression, anxiety, difficulty sleeping), as well as general issues (pain, fatigue, asthenia, headaches) [17].

The following COS were found in the COMET database: PROMS-Cancer Core [25], Cancer Survivorship Core [26], EORTC QLQ-SURV100 [27]. The following PROMs were retrieved through additional hand search: PROMIS-10, PROMIS-PF, FACT-G, FACT-Fatigue, FACT-ES, WHO5, WHO-DAS12, ECOG Performance status, and PRO-CTCAE.

### Expert group (EG) meetings

The results from EG meetings are listed in Table 2. Seventeen EG members included five researchers, five oncologists, two surgeons, two radiation oncologists, one nurse specialist, one patient advocate, and one pharmaceutical industry representative. They represented Germany, The Netherlands, Spain, United Kingdom, United States, Australia, Sweden, Portugal, and Switzerland.

**Table 2:** Results from the expert group meetings

Meeting	Meeting topic	Meeting results
1: Feb 12th, 2021	COS patient population	The target group was defined as patients with metachronous or synchronous MBC in active treatment. By metachronous MBC, we mean MBC developed after initial treatment for early breast cancer; by synchronous MBC, we mean MBC at first diagnosis of breast cancer This did not include end-of-life phase
2: Feb 26th, 2021	Baseline patient characteristics	Sixteen baseline characteristics were selected—eight retrieved from the literature, eight added by the EG
3: Mar 12th, 2021	Treatment modalities and lines	Registration of treatment modalities and lines for MBC were defined, including chemotherapy (with/without targeted therapy) and hormonal therapy (with/without targeted therapy), and treatment line in terms of number the patient currently receives (1, 2–3, 4 and more). We count treatment lines separately for chemotherapy and hormonal therapy
4: Mar 26th, 2021	Survival and disease control	Survival and disease control: included outcomes are *Overall survival* and *Death attributed to breast cancer*
5:Apr 9th, 2021	Adverse treatment effects	Adverse effects of treatment, describing the *Name of complication* and *Severity of complications* for 19 adverse effects, and *Emergency Unit admissions* and *Intensive care admissions*
6: Apr 30th, 2021	HRQoL and functioning	HRQoL and functioning, including 53 outcomes, such as daily functioning, psychosocial, emotional, and sexual functioning

*Description* Results from each Expert group meeting session

*COS* core outcome set, *EG* expert group, *HRQoL* health related quality of life, *MBC* metastatic breast cancer

The EG considered 85 variables relevant. Furthermore, 22 variables were added during the meetings or during pilot testing with patient advocates; 32 variables were excluded, these were already (partially) covered by other items or were beyond of scope. The Delphi started with 115 outcomes *(125 based on literature minus 32 items that were excluded by the EG, plus 22 that were added by the EG; Fig. **2**).*

### Delphi consensus procedure

Participants (*n* = 141) gave consent to participate in a modified Delphi procedure, including 45 patients/patient advocates, 64 HCPs/academic researchers, 28 pharmaceutical industry representatives, and 4 health authority/regulator employees (Supplementary Table 2). The retention rate for each category was 87.2% (*n* = 123): 43 patients/patient advocates (95.6%), 56 healthcare professionals and academic researchers (87.5%), 20 pharmaceutical industry representatives (71.4%), and 4 health authority/regulator employees (100.0%).

In the first round, all stakeholder groups scored 22/115 of the variables as ‘highly relevant’. Furthermore, 19 additional variables were suggested. *In the second round, 134 outcomes were included (all 125 outcomes of the first, plus 19 new outcomes that were added by the Delphi participants in the first round; Fig. **2**).* From these, 60/134 were scored as highly relevant by patients, HCPs/academics, and industry.

### Final consensus meeting

The resulting 74 variables (134 minus 60) for which no consensus was reached, measurement frequencies, and outcome measures were discussed.

Consensus was not reached in the Delphi rounds on *Age, Menopausal status*, *Frailty, Family history of breast cancer, Healthcare access, Risk reducing surgery before diagnosis of metastases, Type of targeted therapy,* and *Activities of daily living.* The EG decided to include these in the final COS since both patients and HCP/researchers considered these outcomes relevant. All 14 adverse events considered relevant by patients were included*: Nausea, Vomiting, Diarrhea, Constipation, Insomnia, Headaches, Shortness of breath/chest tightness, Damage or dysfunction of nerve(s), Weight loss or increase, Sexual/gynaecological symptoms, Fever, High blood sugar (hyperglycemia), High blood pressure (hypertension), Malnutrition*. The EG added three adverse events crucial for monitoring systemic treatments: *Joint pain, Rash, Cough*. They added fourteen HRQoL outcomes considered relevant by patients. This included *(General) Worries and fears, Fear of cancer progression, Worry impact of cancer on children, Uncertainty/unknown future, Worry impact of patient's death on children and family, Autonomy, Ability to work, Financial impact, Problems with insurances; loans; mortgages, Loss of income, Relationship/marital problems, Relationship between patient and medical team, Physical activity, Swelling of arms and legs*.

**Fig. 3:**
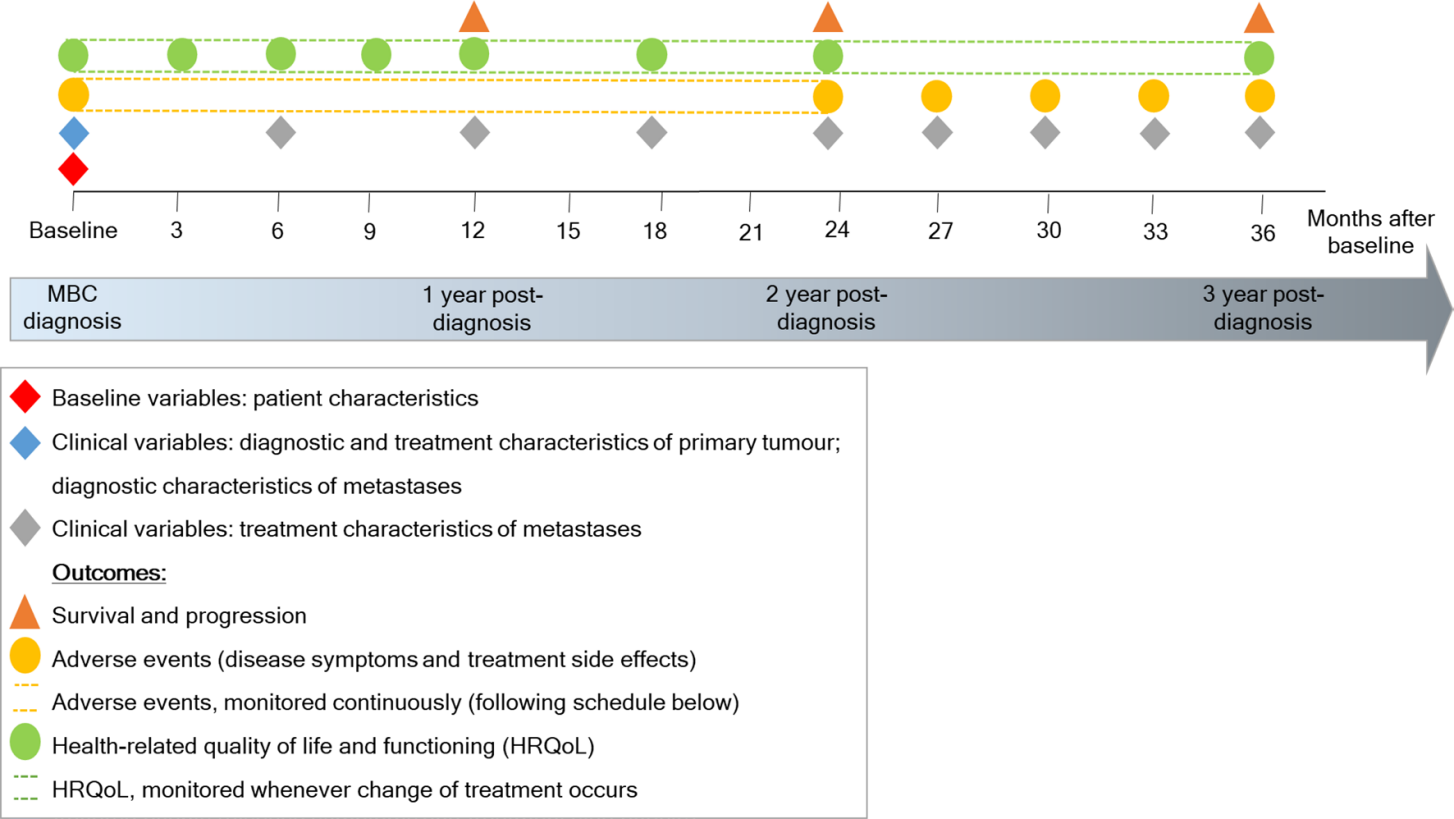
Measurement frequencies per category of outcomes

The final COS consists of 101 variables (Table 3; Supplementary Table 1 includes lay descriptions of each variable). The final COS includes (1) 9 baseline patient characteristics; (2) 42 clinical variables; (3) 50 patient reported outcomes (PROs) about HRQoL and adverse events. Measurement frequencies are presented in Fig. 3.

**Table 3:** Final core outcome set

Outcome	Measurement instrument	Measurement frequency	Population
Variables for data normalisation			
Baseline variables: patient characteristics			
Age (year of birth)	Retrieved automatically from electronic health record or clinician-reported/administrative	At baseline	All patients
Menopausal status			
Activities of daily living / performance status			
Frailty stage			
Family history of breast cancer			
Risk reducing surgery before diagnosis of metastases			
Healthcare access	Patient-reported		
Comorbidities (*including* Other cancer types)	Patient-reported: SACQ		
Clinical variables: diagnostic and treatment characteristics of primary tumour			
Date of histological diagnosis	Retrieved automatically from electronic health record or clinician-reported/administrative	At baseline	In case of metastases developed after initial treatment for early breast cancer (i.e. metachronous metastases)
Type of breast cancer			
Tumour grade			
Clinical cancer stage			
Pathological cancer stage			
Size of invasive tumour			
Number of lymph nodes involved			
Estrogen receptor status			
Progesteron receptor status			
HER-2-status			
(Reconstructive) surgery			
Number of lymph nodes resected			
Chemotherapy			
Radiotherapy			
Hormonal therapy			
Targeted therapy			
No therapy			
Clinical variables: diagnostic and treatment characteristics of metastases			
Date of histological diagnosis of the metastases	Retrieved automatically from electronic health record or clinician-reported/administrative	At baseline	All patients
Oligo metastases/Metastases potentially amenable for local treatment			
Localization of metastases			
Type of the metastases based on breast tissue			
Estrogen receptor status of the metastases			
Progesteron receptor status of the metastases			
HER-2-status of the metastases			
Result of clinical genetic tests			
Start date of new treatment of metastases	Retrieved automatically from electronic health record or clinician-reported/administrative	*First two years after MBC diagnosis:* every 6 months; *Subsequent years:* every 3 months	All patients
Treatment status (treatment of metastases)			
Standard therapy versus experimental/clinical trial therapy			
Time from diagnosis to treatment			
Treatment of metastases: Chemotherapy (with or without targeted therapy)			
Lines of Chemotherapy (with or without targeted therapy)			
Treatment of metastases: Hormonal therapy (with or without targeted therapy)			
Lines of hormonal therapy (with or			
without targeted therapy)			
Treatment of metastases: Radiotherapy			
Localisation of (stereotactic) radiotherapy			
Surgery on primary site			
Surgery on metastatic leasions			
Outcomes			
Survival and progression			
Overall survival	Retrieved automatically from electronic health record or clinician-reported/administrative	Annually	All patients
Death attributable to breast cancer			
Progression Free Survival / duration of response			
Objective response			
Adverse events: disease symptoms and treatment side effects			
Fatigue	Patient-reported: PRO-CTCAE®	*For online administration:* See Fig. 3 for schedule for optimal collection; *For paper administration: First two years after MBC diagnosis:* every cycle for chemotherapy treatment, every month for targeted therapy; *Subsequent years:* every three months	*All patients*
Insomnia			
Cough			
Shortness of breath/chest tightness			
Pain			
Nausea			
Vomiting			
Diarrhea			
Constipation			
Joint pain			
Headaches			
Rash			
Hand-foot syndrome			
Inflamed and sore mouth			
Damage or dysfunction of nerve(s) (neuropathy)			
Fever	CTCAE®		
High blood sugar (hyperglycemia)			
High blood pressure (hypertension)			
Thrombosis			
Malnutrition			
Health-related Quality of Life and functioning			
General well-being / general quality of life	EORTC-QLQ-C30	*If changes in treatment:* At change of treatment *If no changes in treatment: First year after MBC diagnosis:* every 3 months; *Second year after MBC diagnosis:* every 6 months; *Subsequent years:* annually	*All patients*
Daily functioning /role functioning	EORTC-QLQ-C30		
Physical functioning	EORTC-QLQ-C30		
Physical activity	‘are you physically active 30 min a day? answers: yes/no)		
Social functioning	EORTC-QLQ-C30		
Emotional functioning	EORTC-QLQ-C30		
Cognitive functioning	EORTC-QLQ-C30		
Autonomy	EORTC Item library (retrieved from: SURV-100)		
Social support	Question Q956 of EORTC Item library		
Relationship/marital problems	Question Q156 of EORTC Item library		
Sexuality and intimacy	EORTC Item library (Question 108 and 109 from SURV-100)		
Worries and fears	EORTC Item library (Q460, Q178 and Q41)		
Fear of cancer progression	EORTC Item library (Q587)		
Worry impact of cancer on children	EORTC Item library (Question 87 of SURV-100)		
Worry impact of patient's death on children and family	EORTC Item library(Q299)		
Overall Symptom Experience/overall bother from side effects	EORTC Item library (Q168)		
Swelling of arms and legs	EORTC Item library (Q916)		
Weight loss or increase	EORTC Item library (Q123 and Q124)		
Uncertainty/unknown future	EORTC Item library (Q42)		
Symptom awareness	EORTC Item library ( Q168) and question 72 of EORTC SURV100 (adapted)		
Financial impact	EORTC-QLQ-C30		
Loss of income	EORTC Item library (Question 92 of EORTC SURV100)		
Ability to work	EORTC Item library (Question 90, 91 and 93 of EORTC SURV100)		
Problems insurances; loans; mortgages	EORTC Item library (Question 88 of EORTC SURV100)		
Relationship between patient and medical team	EORTC item library (Q145, Q562)		

*Description* Suggested measurement instruments and measurement frequencies for each variable/outcome included in the final core outcome set. Since the resources and capacity to adopt and implement the COS in clinical setting differ between as well as within countries, we constructed recommendations for the range of situations that may occur in different settings

*MBC* metastatic breast cancer, *SACQ* self-administered comorbidity questionnaire, *PRO-CTCAE* patient-reported outcomes version of the common terminology criteria for adverse events

## Discussion

We defined a consensus-based COS for patients with MBC at first diagnosis (de novo MBC) or who developed it after initial treatment for early breast cancer (recurrent MBC)—101 variables cover: (1) baseline patient characteristics, (2) clinical variables, and 3) HRQoL and adverse events. The COS will be distributed, its implementation promoted, and its feasibility studied through the Innovative Medicines Initiative Health Outcomes Observatory (H2O) project (health-outcomes-observatory.eu) [28]. This internationally standardized COS will allow measurement consistency and avoid outcome-reporting bias in disease management, providing a tool to be homogenously used on most clinical trials, allowing for better interpretation of results and better research-informed patient and policy decisions [13]. Its implementation has the potential to change clinical encounters for MBC patients by improving symptom management and patient-provider communication [29–31].

In general, the PROMs movement has largely been driven by research agendas or service payers goals, without focusing effectively on improving HRQoL from the patient’s perspective [32]. Furthermore, there is a lack of widespread measurement of patient outcomes needed to support patient-centered MBC care [8]. As such, they may not fully capture patients’ experiences of disease and its impact on their lives. Not all study participants expressed equal relevance for the eventually selected adverse events and HRQoL outcomes. Since this COS explicitly aims to capture the experience of MBC patients with disease and treatment, we included all variables that patients considered relevant. This led to the inclusion of 14 adverse events and 14 HRQoL outcomes additional to the outcomes agreed upon in the Delphi. Outcomes that are especially important and specific to the experiences of MBC patients were selected, including fears and worries of the disease impact on children, family, work, and the future. The importance of measuring these psychosocial outcomes have been highlighted in literature [5, 9, 10] and makes our COS particularly comprehensive.

Gaps in information and support for MBC patients have been highlighted at the 2019 ABC Global Alliance Annual Meeting. They concluded that patients’ individual circumstances, values, needs, and fears often felt unnoticed and thus, the patient-healthcare provider relationship could be improved. Furthermore, the ABC report stated that many patients expressed a desire for a greater role in the decision-making process [33]. Our COS includes an item on the *Relationship between patient and medical team* that could start the conversation in clinical practice about both gaps. Even though this item is technically a patient-reported experience measure (PREM), the impact of the patient-provider relationship may have a profound impact on disease experience and HRQoL [33, 34] and the EG therefore agreed to include it.

### Strengths and weaknesses

Strengths of our study include strong patient representation, broad multi-stakeholder involvement, and wide international outreach. Patients/patient representatives made up a third of participants in the Delphi, and cancer patient organizations in Austria, Germany, Netherlands, Spain, and Portugal were consulted during all steps of this study, as well as through the involvement of the ABC Global Alliance [35]. Besides patients, industry and care delivery perspectives representing fifteen countries were brought together, which is uncommon in COS development. Also, the Delphi was hosted in six languages, contributing to wide outreach. This inclusive engagement approach can drive broad acceptance and wide adoption of this COS.

The COS includes 101 variables, fully capturing the patient-relevant experience of care for the MBC population and covering the latest innovative therapeutic modalities and targeted treatments. The downside is the potentially high registration burden for patients. Fifty outcomes are captured through PROMs; the other variables could be obtained from electronic health records or clinical registries. To reduce patient burden, we recommend that 26 PROs are measured only once every few months (category ‘Health-related Quality of Life and functioning’; measured at every change of treatment (i.e. at disease progression), or, if the disease is stable, year 1: every 3 months; year 2: every 6 months; and annually in subsequent years. This leaves 24 PROs to be completed more frequently (category: ‘Adverse events: disease symptoms and treatment side effects’; measurement frequency dependent on treatment trajectory, emphasizing the need to evaluate when there is disease progression or intolerable toxicity). The patients’ efforts will however give them in return the ability to track their symptoms over time, to gain feedback on their HRQoL and the impact of treatment on it, and to improve communication with their HCPs [29–31].

The COS will be available in English, Spanish, Portuguese, and German and is thus suitable for implementation in clinical practice across geographies. However, the resources and capacity to adopt and implement the COS in clinical setting differ between as well as within countries, including unequal access to care and availability of treatments [8, 33]. We have therefore constructed recommendations for the range of situations that may occur in different settings. For instance, we envisaged situations in which baseline characteristics are retrieved automatically from the electronic health record and PROMs are administered through online applications. The other end of the spectrum exists of paper health records and paper-and-pencil completed PROMs surveys. For the latter, we defined different frequency schedules. We used a certain pragmatism in selecting outcomes during the final consensus meeting, bearing in mind what is feasible to measure and difficulties in collecting the data in practice. Still, the implementation of any COS requires a supportive environment and a strong mandate to reach successful implementation.

The ABC-5 describes how specific PROMs for evaluating HRQoL in MBC patients are missing and should be developed [8], confirmed by Clarijs et al. [16]. We have coordinated our efforts with the currently ongoing development of the EORTC Quality of Life Questionnaire module for MBC [17]; this will be the first MBC-dedicated PROM, which use we would recommend upon becoming available. This work is strongly recommended by the ABC Global Alliance, a multi-stakeholder platform where more than 190 organizations worldwide collaborate to develop and share resources aiming at improving the lives of advanced/metastatic breast cancer [35]. Lastly, this outcome set was accredited by ICHOM, which is a recognition of our COS as leading example in value based health care and will boost implementation in clinical practice.

### Clinical practice recommendations and future research

H2O will (initially) implement the COS in Austria, Germany, the Netherlands, and Spain. They will coordinate the efforts in providing patients with digital tools to report their health outcomes in a standardized way [28]. It will generate output that is relevant for patients, clinical research, and clinical care, leading to collecting ‘real-world data’. Direct value is added for patients by improving symptom management and patient-provider communication [29–31]. The collection of real-world data complements current existing clinical trial data; it could bridge the current gaps in epidemiologic and outcomes research [8, 33], leading to insights about treatments and HRQoL and adverse events outside of strictly selected trial populations. These insights will add to achieving best possible HRQoL and maximizing survival for MBC patients, further improving care.

Future research will focus on the implementation feasibility in clinical practice and suitability of measurement instruments, with possible development of specific measurement strategies distinctive by metastasis localization (breast, bone, visceral) or genetic differences. The COS describes an overview of outcomes relevant for the broad population of MBC patients that should minimally be measured and reported in clinical practice and clinical trials, and was not specified for sub-populations of patients. With advances in treatment strategies for MBC, the COS must evolve over time and be re-evaluated. Other developments could be slimming down the COS by selecting parameters that are critical in driving healthcare decisions, or adapting the COS through Computerized Adaptive Testing (CAT), which could reduce registration burden [36]. Last, the widespread implementation of this COS could lead to a more homogeneous collection of HRQoL data in clinical trials [13]. Currently, trials use different tools, making data interpretation very difficult and leading to conclusions disconnected from clinical reality.

### Conclusion

An international multi-stakeholder group of 141 MBC patients and experts defined a COS for MBC. This COS will enable capturing the patient perspective of the impact of cancer and its treatments through combined administrative data, clinical records, clinician-reported measures, and PROMs, in an internationally standardized way.

## Supplementary Information

Below is the link to the electronic supplementary material.

Supplementary file1 (DOCX 24 kb)

## Data Availability

The data dictionary of the COS is available through ICHOM connect (https://connect.ichom.org/).
