# Supplementary material for: International development of a patient-centered core outcome set for assessing health-related quality of life in metastatic breast cancer patients
Source: Breast Cancer Res Treat. 2023 Jan 20;198(2):265–81. doi: 10.1007/s10549-022-06827-6 (PMC10020292; doi:10.1007/s10549-022-06827-6)
Supplement: Supplementary file 1 — Supplementary file1 (DOCX 24 kb) [file 10549_2022_6827_MOESM1_ESM.docx]

**Supplementary materials for:**

**International development of a patient-centered core outcome set for assessing health-related quality of life in metastatic breast cancer patients**

K.M. de Ligt^1^, PhD, B.H. de Rooij^2,3^, PhD, E. Hedayati^4,5^, PhD, M.M. Karsten^6,7^, M.D., V.R. Smaardijk^2^, PhD, M. Velting^8^, MSc, C. Saunders^9^, PhD, L. Travado^10^, PhD, F. Cardoso^10^, MD, E. Lopez^11^, PhD, N. Carney^12^, PhD, Y. Wengström^4,13^, PhD, A. Ives^14^, PhD, G. Velikova^15,16^, PhD, M. D. L. Sousa Fialho^17^, MSc, Y. Seidler^18^, Dr., T.A. Stamm^18,19^, PhD, L.B. Koppert^20^, PhD, L.V. van de Poll-Franse^1,2,3^, PhD.

**Corresponding Author:** Kelly de Ligt, PhD.
Netherlands Cancer Institute, Division of Psychosocial Research and Epidemiology
PO Box 90203, 1006 BE Amsterdam
e-mail: k.d.ligt@nki.nl | telephone: +31 20 512 9111

**Submitted to:** Breast Cancer Research and Treatment

**Supplementary table 1: Final core outcome set**

| **Outcome** | **Description** |
| --- | --- |
| **Patient characteristics** |  |
| Age (year of birth) | Age (at time of diagnosis) |
| Menopausal status | Whether a woman still has monthly cycles. 'Premenopausal' describes women who experience menstrual bleeding; 'postmenopausal' describes women who have not experienced menstrual bleeding >12 months, in the absence of any surgery or medical condition that may cause bleeding to artificially stop (use of hormonal birth control, overactive thyroid, etc.)). |
| Comorbidities (*including* Other cancer types) | Living with more than one chronic disease or health condition |
| Activities of daily living / performance status | Ability to perform certain activities of daily living (ADL) (e.g. eating, dressing, washing, toilet use, household activities, managing money) without the help of others |
| Frailty stage | A weakened health state related to ageing (e.g. reduced physical condition and cognitive decline). Frail patients may have an increased burden of symptoms and a reduced tolerance to treatment. |
| Healthcare access | Able to access main care giving team any time and day of the week |
| Family history of breast cancer | Immediate family member who has or had breast cancer |
| Risk reducing surgery before diagnosis of metastases | Received surgical removal of organs at high risk of developing cancer (e.g. removal of the breasts) prior to metastases |
| **Tumour characteristics*** |  |
| Date of histological diagnosis | Date on which the breast cancer diagnosis is confirmed through punction of the breast. |
| Type of breast cancer | Breast cancer sub-classification based on tissue examination (histology) (applicable for the primary tumour in case of metachronous metastases) |
| Tumour grade | Classification of the appearance of tumour cells under the microscope. Low-grade tumours (with cells very similar to healthy cells) are often referred to as well-differentiated, while high-grade tumours (with strictly abnormal cells) are called poorly differentiated or undifferentiated. |
| Clinical cancer stage | Cancer stage (i.e. the extent to which a cancer has developed by growing and spreading) based on results of tests done before surgery, such as physical examinations and imaging scans. Per UICC / IASLC / AJCC 8th edition. |
| Pathological cancer stage | Cancer stage (i.e. the extent to which a cancer has developed by growing and spreading) based on what is found during surgery. Per UICC / IASLC / AJCC 8th edition. |
| Size of tumour | Size of the tumour. |
| Number of lymph nodes involved | Number of lymph nodes with positive findings for breast cancer. |
| Oestrogen receptor status | Whether the cancer may receive signals from the hormone oestrogen that could promote the growth of cancer cells. |
| Progesterone receptor status | Whether the cancer may receive signals from the hormone progesterone that could promote the growth of cancer cells. |
| HER-2-status | Whether the cancer has certain characteristics that could promote the growth of cancer cells, in this case a protein called Human epidermal growth factor receptor 2 (HER-2). Tumours with HER-2 are usually treated with therapy that specifically identifies and targets this protein (i.e. targeted therapy). |
| **Treatment characteristics*** |  |
| (Reconstructive) surgery | Received type of cancer treatment in which the tumour and nearby tissue are removed, sometimes with removal of all breast tissue (mastectomy). With or without breast reconstruction (one or both breasts are restored). |
| Number of lymph nodes resected | Number of lymph nodes surgically removed. |
| Chemotherapy | Received type of cancer treatment that uses drugs to kill cancer cells. |
| Radiotherapy | Received type of cancer treatment that uses high doses of radiation to kill cancer cells and shrink tumours (applicable for the primary tumour in case of metachronous metastases) |
| Hormonal therapy | Received type of cancer treatment that uses drugs to slow or stop the growth of cancer that grows under the influence of hormones. |
| Targeted therapy | Received type of cancer treatment that identifies biomarkers in genetic or other kinds of tests to precisely identify and kill certain types of cancer cells (i.e. “personalized medicine” or “precision medicine”). |
| No therapy | Received no cancer therapy that is part of medical care. |
| **Characteristics of metastases*** |  |
| Date of histological diagnosis of the metastases | Date on which the diagnosis of metastasized breast cancer is confirmed (If there is a biopsy, than date of biopsy is leading. If there is only a date of imaging, then this date is leading. If both, date of biopsy is leading). |
| Oligo metastases/Metastases potentially amenable for local treatment | When only a limited number of metastases is found in the body (up to 5 and not necessarily in the same organ), that have a limited size, metastatic breast cancer can potentially be treated with surgery. By this, surgeons aim to remove all metastatic sites in the body. The goal is to completely remove the breast cancer from the body. *(Official 5th ESO-ESMO international consensus guidelines for advanced breast cancer definition: Oligo metastatic disease is defined as low volume metastatic disease with limited number and size of metastatic lesions (up to 5 and not necessarily in the same organ), potentially amenable for local treatment, aimed at achieving a complete remission status.)* |
| Localisation of metastases | Localization of the metastases in the body (e.g. bone, visceral [= in the intestines, independent of how many organs] or brain) |
| Type of the metastases based on breast tissue | Type of the metastases determined based on tissue examination (histology): in lining of the milk ducts (ductal) or milk-producing glands (lobular) |
| Oestrogen receptor status of the metastases | Whether the metastasis has receptors for oestrogen which could promote the growth of cancer cells |
| Progesterone receptor status of the metastases | Whether the metastasis has receptors for progesterone which could promote the growth of cancer cells |
| HER-2-status of the metastases | Whether the metastasis has a protein called Human epidermal growth factor receptor 2 (HER-2) which could promote the growth of cancer cells |
| Result of clinical genetic tests | Result of clinical genetic tests (BRCA1/2, CHEK2 1100delC, ATM, PALB2, PTEN, NF1, or 'not applicable') |
| **Treatment of metastases*** |  |
| Start date of new treatment of metastases | Date when new treatment line (in case of chemotherapy or hormonal therapy) or treatment modality (in case of radiotherapy and surgery) was started. |
| Treatment status (treatment of metastases) | Status of treatment of the metastases (i.e. treatment continued as planned, stopped for adverse events, stopped for progression, stopped for refusal patient, patient deceased) |
| Standard therapy versus experimental/clinical trial therapy | Treatment received according to the guidelines, standard therapy other than guideline or as part of a clinical trial |
| Time from diagnosis to treatment | Time between date of diagnosis and start date of first treatment |
| Treatment of metastases: Chemotherapy (with or without targeted therapy) | Type of cancer treatment that uses drugs to kill cancer cells (with or without targeted therapy, i.e. drugs to precisely identify and kill certain types of cancer cells) |
| Lines of Chemotherapy (with or without targeted therapy) | Which line of chemotherapy (in terms of number) the patient currently receives: 1, 2-3, 4 and more. Only applicable for systemic therapy. We consider lines of treatment separately for chemotherapy and hormonal therapy. |
| Treatment of metastases: Hormonal therapy (with or without targeted therapy) | Type of cancer treatment that uses drugs to slow or stop the growth of cancer that grows under the influence of hormones (with or without targeted therapy, i.e. drugs to precisely identify and kill certain types of cancer cells) |
| Lines of hormonal therapy (with or without targeted therapy) | Which line of hormone therapy (in terms of number) the patient currently receives: 1, 2-3, 4 and more. Only applicable for systemic therapy. We consider lines of treatment separately for chemotherapy and hormonal therapy. (with or without targeted therapy, i.e. drugs to precisely identify and kill certain types of cancer cells) |
| Treatment of metastases: Radiotherapy | Type of cancer treatment that uses high doses of radiation to kill cancer cells and shrink tumours. Stereotactic (precisely-targeted radiation in fewer high-dose treatments) and/or palliative (aims to shrink but not cure the cancer) |
| Localisation of (stereotactic) radiotherapy (Optional) | Localisation of (stereotactic) radiotherapy |
| Surgery on primary site | Type of cancer treatment that resects the tumour and nearby tissue on the site of primary tumour (part of the body where the cancer started) |
| Surgery on metastatic lesions | Type of cancer treatment that resects the metastases and nearby tissue on metastatic lesions (parts of the body to where the cancer has spread) |
| **Survival and progression** |  |
| Overall survival | Length of time (in days) that a patient remains alive from the date of diagnosis of metastasized breast cancer |
| Death attributed to breast cancer | Whether death was attributed to breast cancer |
| Progression Free Survival | The length of time during and after the treatment of breast cancer, that a patient lives with the breast cancer but it does not get worse |
| Objective response rate (ORR) | The assessment of the tumour burden after a given treatment. It demonstrates the efficacy of a treatment. |
| Duration of Response | The length of time that a tumour continues to respond to treatment without the cancer growing or spreading |
| **Acute complications** |  |
| Fatigue | Feeling weak, lacking in energy, tired, drained, or exhausted |
| Insomnia | E.g. problems falling asleep, waking up frequently at night, trouble sleeping, waking up too early |
| Cough | Problems with coughing (e.g. dry cough, wet cough, coughing up blood) |
| Shortness of breath/chest tightness | Shortness of breath or respiratory problems, which may happen at rest, and may require treatment |
| Pain | Unpleasant physical sensation that limits activities of daily life, limits self-care and/or requires medication or hospitalisation (e.g. arching joints) |
| Nausea | Feeling of being sick, which may lead to impact on intake of food and/or fluids and/or normal activities |
| Vomiting | Vomiting |
| Diarrhoea | Passing looser stools or passing stools more often than usual |
| Constipation | Having difficulty passing stools, which may be large and hard |
| Joint pain | Joint pain (e.g. hip and back) |
| Headaches | Pain localized in and around the head |
| Rash | Discolorations and skin changes (vesicles, nodules, pustules) of various sizes and groupings on the skin and mucous membranes |
| Hand-foot syndrome | Rash consisting of painful, swollen, dry, red-coloured, itchy hands and feet (often with tight, shiny skin) that may have blisters |
| Inflamed and sore mouth | Inflammation of the entire oral mucosa and often also of the lips |
| Damage or dysfunction of nerve(s) | Damage or dysfunction of one or more nerves that typically results in numbness, tingling, muscle weakness and pain in hands and/or feet. |
| Fever | An increase in body temperature above 38 degrees Celsius |
| High blood sugar (hyperglycaemia) | The blood glucose level is too high |
| High blood pressure (hypertension) | High blood pressure |
| Thrombosis | Blood clots block veins or arteries |
| Malnutrition | Deficiencies, excesses, or imbalances in a person's intake of energy and/or nutrients |
| **HRQoL** |  |
| General well-being | A general indicator of overall health and quality of life |
| General quality of life | A general indicator of life satisfaction and physical, emotional, and social well-being |
| Daily functioning /role functioning | The ability to perform and participate in daily activities, i.e. care for oneself, physical ability, working ability, including functioning in usual social and occupational roles |
| Physical functioning | Ability to perform physical activities (e.g. trouble doing strenuous activities, long walk/short walk; stay in bed/chair during the day; need help with eating, dressing, washing yourself or using the toilet) |
| Physical activity | E.g. walking, cycling, swimming |
| Social functioning | The extent to which the patient's physical condition or medical treatment has affected family life and social activities (e.g. theater, hobbies, sports) |
| Emotional functioning | Degree of emotional functioning (e.g. feeling tense, worrying, feeling irritable, feeling depressed, feeling sad, showing empathy to other people) |
| Cognitive functioning | Loss of cognitive abilities such as remembering things, concentrating, thinking, keeping focus on tasks, or performing multiple tasks simultaneously |
| Autonomy | Ability of a person to make his or her own decisions |
| Social support | The degree in which a person feels supported by his/her network, including through emotional support, tangible support, affectionate support, and positive social interactions |
| Relationship/marital problems | Feeling that partner relation improved or deteriorated, being able to talk about your illness with your partner or the person closest to you |
| Sexuality and intimacy | Sexual desire, sexual activity, pleasure during sex |
| Worries and fears | Worries and fears related to breast cancer, dying from breast cancer or health in the future |
| Fear of cancer progression | Fear that cancer could progress or spread to another part of the body |
| Worry impact of cancer on children | Concerns about the impact of cancer on the children of the cancer patient |
| Worry impact of patient's death on children and family | Concerns about the impact of the cancer patient's death on the cancer patient's children and family |
| Overall Symptom Experience/overall bother from side effects | The extent to which someone is bothered by side effects from cancer and the associated disruption to normal activities |
| Swelling of arms and legs | Swelling of arms and legs |
| Weight loss or increase | Weight loss or increase |
| Uncertainty/unknown future | Concerns about the impact of cancer on the future, making the (nearby) future uncertain |
| Symptom awareness | Being alert for symptoms/side effects and contacting doctor for this |
| Financial impact | The impact of cancer on financial aspects of life |
| Loss of income | Loss of income due to cancer |
| Ability to work | The experienced degree of limitation in doing work |
| Problems insurances; loans; mortgages | Problems with taking out insurances, loans, and/or mortgages due to cancer and coverage of the daily medical needs such as treatment and medication due to cancer. |
| Relationship between patient and medical team | Relationship between patient and medical team, built on trust, respect, communication, and a common understanding of both the medical team' and patients' sides. |

**Abbreviations:
HRQoL:** Health-related quality of life

**Supplementary Table 2: characteristics of participants in the Modified Delphi procedure**

|  | | **Patients/**  **patient advocates** | **HCP/**  **academic researchers** | **Industry** | **HA/HR** | **Total** |
| --- | --- | --- | --- | --- | --- | --- |
| Participants (N) | | **45** | **64** *(38 HCP, 26 researchers)* | **28** | **4** | **141** |
| Mean age (in years) | | 56.4 | 43.2 | 45.1 | 43.3 | 47.5 |
| Country of residence | |  |  |  |  |  |
|  | Argentina | 0 | 1 | 2 | 0 | 3 |
|  | Australia | 13 | 2 | 0 | 0 | 15 |
|  | Austria | 1 | 2 | 1 | 2 | 6 |
|  | Belgium | 0 | 1 | 0 | 0 | 1 |
|  | Canada | 0 | 0 | 1 | 0 | 1 |
|  | Germany | 0 | 12 | 2 | 0 | 14 |
|  | Italy | 0 | 3 | 3 | 0 | 6 |
|  | Netherlands | 9 | 15 | 4 | 1 | 29 |
|  | Portugal | 2 | 7 | 2 | 0 | 11 |
|  | Spain | 7 | 6 | 1 | 1 | 15 |
|  | Sweden | 13 | 9 | 4 | 0 | 26 |
|  | Switzerland | 0 | 0 | 3 | 0 | 3 |
|  | Turkey | 0 | 0 | 1 | 0 | 1 |
|  | United Kingdom | 0 | 6 | 1 | 0 | 7 |
|  | United States of America | 0 | 0 | 3 | 0 | 3 |
| Gender | |  |  |  |  |  |
|  | Male | 0 | 10 | 10 | 2 | 22 |
|  | Female | 44 | 54 | 18 | 2 | 118 |
|  | I would rather not say | 1 | 0 | 0 | 0 | 1 |
| Involvement in H2O project | |  |  |  |  |  |
|  | Yes | 8 | 23 | 10 | 0 | 41 |
|  | No | 37 | 41 | 18 | 4 | 100 |
| **Abbreviations:**  **HA/HR:** Health authority/Health regulator  **HCP:** health care providers  **H2O:** Health Outcomes Observatory, project by the Innovative Medicines Initiative. | | | | | | |
